# The impact of chronic disease self-management programs: healthcare savings through a community-based intervention

**DOI:** 10.1186/1471-2458-13-1141

**Published:** 2013-12-06

**Authors:** SangNam Ahn, Rashmita Basu, Matthew Lee Smith, Luohua Jiang, Kate Lorig, Nancy Whitelaw, Marcia G Ory

**Affiliations:** 1 School of Public Health, Division of Health Systems Management and Policy, The University of Memphis, Memphis, TN, USA; 2 Scott & White Healthcare, Temple, TX, USA; 3 Department of Health Promotion and Behavior, College of Public Health, The University of Georgia, Athens, GA, USA; 4 Department of Epidemiology and Biostatistics, School of Rural Public Health, Texas A&M Health Science Center, College Station, TX, USA; 5 Department of Medicine, Stanford University, Palo Alto, CA, USA; 6 National Council on Aging, Washington, DC, USA; 7 Department of Health Promotion and Community Health Sciences, School of Rural Public Health, Texas A&M Health Science Center, College Station, TX, USA

**Keywords:** Chronic disease self-management program, Healthcare utilization, Healthcare cost savings

## Abstract

**Background:**

Among the most studied evidence-based programs, the Chronic Disease Self-Management Program (CDSMP) has been shown to help participants improve their health behaviors, health outcomes, and reduce healthcare utilization. However, there is a lack of information on how CDSMP, when nationally disseminated, impacts healthcare utilization and averts healthcare costs. The purposes of this study were to: 1) document reductions in healthcare utilization among national CDSMP participants; 2) calculate potential cost savings associated with emergency room (ER) visits and hospitalizations; and 3) extrapolate the cost savings estimation to the American adults.

**Methods:**

The national study of CDSMP surveyed 1,170 community-dwelling CDSMP participants at baseline, 6 months, and 12 months from 22 organizations in 17 states. The procedure used to estimate potential cost savings included: 1) examining the pattern of healthcare utilization among CDSMP participants from self-reported healthcare utilization assessed at baseline, 6 months, and 12 months; 2) calculating age-adjusted average costs for persons using the 2010 Medical Expenditure Panel Survey; 3) calculating costs saved from reductions in healthcare utilization; 4) estimating per participant program costs; 5) computing potential cost savings by deducting program costs from estimated healthcare savings; and 6) extrapolating savings to national populations using Census data combined with national health statistics.

**Results:**

Findings from analyses showed significant reductions in ER visits (5%) at both the 6-month and 12-month assessments as well as hospitalizations (3%) at 6 months among national CDSMP participants. This equates to potential net savings of $364 per participant and a national savings of $3.3 billion if 5% of adults with one or more chronic conditions were reached.

**Conclusions:**

Findings emphasize the value of public health tertiary prevention interventions and the need for policies to support widespread adoption of CDSMP.

## Background

People living with prevalent chronic conditions such as heart disease, cancer, diabetes, stroke, and chronic lung disease account for 75% of healthcare expenditures in the United States [1]. This disease burden profile will exacerbate with the rapid aging of the American population. The number of Americans with chronic conditions is projected to increase by 37% (i.e., 46 million people) from 2000 to 2030 [2].

The Patient Accountability and Affordable Care Act underscores the potential of chronic disease management for improving the efficiency of healthcare in the U.S. This Act also recognizes the importance of community. Helping people develop the knowledge, skills, and motivation needed to make healthier choices for better self-management is seen as an essential part of the national prevention strategy [3]. Toward this end, there is growing evidence that structured small group chronic disease self-management education, such as Stanford’s Chronic Disease Self-Management Program (CDSMP), can help participants improve their health behaviors, health outcomes, and reduce healthcare utilization [4,5]. Although it has been more than 10 years since positive findings from the initial randomized trial were reported, there is no current information on how healthcare utilization and associated healthcare costs can be averted by a nationwide dissemination of CDSMP.

Thus, the current study examines reductions in healthcare utilization among CDSMP participants to identify potential cost savings to the health system as a result of participating in the program. Three specific purposes of this study were to: 1) document reductions in healthcare utilization among participants of the national study of CDSMP; 2) calculate potential cost savings associated with emergency room (ER) visits and hospitalizations using age-adjusted national cost estimates; and 3) extrapolate the cost savings estimation to the American adults with one or more chronic conditions. Drawing upon findings from a parallel nationwide CDSMP study [6], the current study proposes to inform policy makers, state officials, healthcare providers, and community agencies about potential healthcare cost savings.

## Methods

CDSMP is an evidence-based, peer-led program consisting of six sessions hosted over six consecutive weeks that empowers participants to develop skills necessary for medical, social role, and emotional management of chronic conditions [4]. It is a public health intervention delivered by trained facilitators in community-based settings throughout the U.S. and around the globe. CDSMP workshops are supported by various federal, state, and local sources as well as healthcare organizations and community agencies. As a translational research study, the National Study of CDSMP surveyed 1,170 community-dwelling CDSMP participants at baseline, 6 months, and 12 months from 22 organizations in 17 states. Approximately 77% (n = 903) and 71% (n = 825) of the 1,170 participants completed the 6-month and 12-month assessments, respectively [6,7]. Participants who completed assessments at both time points tended to be older, and completers of the 6-month assessment were more likely to be non-Hispanic white [6]. Self-reported data using validated questionnaires were collected about health conditions, health behaviors, and healthcare utilization from a multi-ethnic population (55.2% were non-Hispanic white) [8].

The procedure used to estimate potential cost savings started by examining the pattern of healthcare utilization among CDSMP participants from self-reported healthcare utilization (i.e., ER visits and hospitalizations) assessed at baseline, 6 months, and 12 months. Generalized mixed effects models (using Stata *gllamm* procedure; [9]) were used to assess change for any ER visits and hospitalizations (binary) from baseline to 6- and 12-month assessments controlling for age, sex, race/ethnicity, education, and number of chronic conditions. Demographic statistics and coding schemes for these variables are documented previous studies [6,7]. These mixed effects models used likelihood-based approaches to provide unbiased estimates of the intervention effects assuming that responses are missing at random.

Once the patterns of health care were documented, the following procedure were undertaken to examine cost issues: 1) calculating age-adjusted average costs for persons with at least one chronic condition using weighted average costs (using MEPS sampling weights) for ER visits and hospitalizations from the Household Component of the 2010 Medical Expenditure Panel Survey (MEPS) [10]; 2) calculating costs saved from reductions in healthcare utilization adjusting for population distribution for three age groups (18–44, 45–64, and 65+); 3) estimating per participant program costs based on expert opinion from program developers and budget calculations from agencies offering the program; 4) computing potential cost savings by deducting program costs from estimated healthcare savings; and 5) extrapolating savings to national populations using Census data [11] combined with national health statistics. Institutional Review Board approval was obtained through Texas A&M University.

## Results

Table 1 shows changes in ER visits from baseline (18%) to 6 months (13%) to 12 months (13%). The odds of ER visits in the past 6 months among CDSMP participants was significantly reduced from baseline to 6-month (Odds Ratio [OR] = 0.68, p = 0.007) and 12-month (OR = 0.68, p = 0.009) assessments controlling for potential confounding factors. Table 1 also shows changes in hospitalizations from baseline (14%) to 6 months (11%) to 12 months (14%). The adjusted odds of hospitalizations was significantly reduced from baseline to 6-month assessment (OR = 0.70, p = 0.025).

**Table 1:** **Adjusted**^**a **^**ratios between baseline and follow-up means for ER visits and Hospitalizations among CDSMP participants (N = 1,170)**

	**%**^**b**^			**Adjusted**^**c **^**change from baseline to 6-month**		**Adjusted change**^**d **^**from baseline to 12-month**	
	**Baseline (n = 1,170)**	**6-Month (n = 903)**	**12-month (n = 825)**	**Adjusted ratio change**	**P-value**	**Adjusted ratio change**	**P-value**
Any ER visit	18%	13%	13%	0.68	0.007	0.68	0.009
Any hospitalization	14%	11%	14%	0.70	0.025	1.02	0.920

^a^All changes are adjusted for sex, age, race/ethnicity, education, and number of chronic conditions.

^b^Raw percentage at baseline, 6-month, and 12-month.

^c^Adjusted odds ratio of any ER visit and hospitalization between baseline and 6-month from the logistic regression models.

^d^Adjusted odds ratio of any ER visit and hospitalization between baseline and 12-month from the logistic regression models.

Table 2 describes calculated cost savings associated with ER visits and hospitalizations among the national study of CDSMP participants. As shown in Table 1, significant reductions in ER use were observed at both the 6-month and 12-month assessments (5% at each time point). There was a significant reduction for hospitalizations at 6 months (3%). The estimated average cost for ER visits and hospitalizations of those having at least one chronic condition from 2010 MEPS data were $1,513 and $18,750, respectively. Total estimated health care costs averted per participant were calculated as $713.80.

**Table 2:** Cost savings estimation based on 2010 national CDSMP data and 2010 Medical Expenditure Panel Survey (MEPS)

**Potential cost savings related to ER visits and hospitalizations among CDSMP participants**					
Potential annual health care savings per CDSMP participant (A)			$713.80		
Age-adjusted cost of ER visits among those having at least 1 chronic condition (CC)^a^	$1,513				
(Baseline = 18%; 6 month post = 13%)	5%	Reduction of ER visits among CDSMP participants in the 1st 6-months	$75.65		
(Baseline = 18%; 12 month post = 13%)	5%	Reduction of ER visits among CDSMP participants in the 2nd 6-months	$75.65		
Age-adjusted cost of hospitalizations among those having at least 1 CC^a^	$18,750				
(Baseline = 14%; 6 month post = 11%)	3%	Reduction of hospitalizations among CDSMP participants in the 1st 6-months	$562.50		
(Baseline = 14%; 12 month post = 14%)	0%	Reduction of hospitalizations among CDSMP participants in the 2nd 6-months	$0.00		
		Estimated number of CDSMP participants (B)	6	10	16
		Estimated CDSMP workshop cost^b^ (C)	$3,500.00	$3,500.00	$3,500.00
Estimated average CDSMP costs per person varying by number of CDSMP participants and workshop costs (D = C÷B)^b^			$583.33	$350.00	$218.75
Net cost savings per CDSMP participant (E = A-D)			$130.47	$363.80	$495.05
**Extrapolation to national savings using Census data combined with MEPS data**					
Number of Americans aged 18 and older from 2010 Census data (F)	234,564,071				
Estimated % of Americans having at least 1 CC^a^ (G)	77%				
Number of Americans aged 18 and older having at least 1 CC (H = F × G)	180,614,335				
		Estimated number of CDSMP participants	6	10	16
			Billion dollars		
National health care savings if we could reach	100%	of people having at least 1 CC (E × H)	$23.6	$65.7	$89.4
	10%		$2.4	$6.6	$8.9
	5%		$1.2	$3.3	$4.5
	1%		$0.2	$0.7	$0.9

*Note.*^a^Based 2010 MEPS; ^b^Based on reported data from two CDSMP national studies in the states of Oregon and Florida and expert’s opinions including CDSMP developers.

Table 2 also displays variation in the average per participant cost of CDSMP by number of enrolled participants per workshop. Assuming a $3,500 per workshop cost, the estimated per participant cost ranged from $583.33 (i.e., 6 participants) to $350 (i.e., 10 participants) to $218.75 (i.e., 16 participants). In a previous national study of 145 CDSMP workshops, the workshops had an average size of 12.7 (±4.18), with the majority of workshops (66.2%) having between 8 and 16 participants [12]. There were small extremes at both ends with 17.9% of the workshops having less than 8 participants and 15.9% having more than 16 participants [12]. When assuming $350 per participant cost based on best estimates from experts and field reports, potential cost savings were estimated to be $363.80 per person (i.e., $713.80 - $350.00). Extrapolating these savings to the national level, potential savings of $65.7 billion could be achieved if CDSMP reached all individuals with one or more chronic condition. More feasibly, potential savings of $3.3 billion could be achieved if the program reached only 5% of this population (approximately 9 million people) or $0.7 billion reaching 1% of this population (approximately 1.8 million people). When assuming $218.75 per participant cost, national healthcare savings were estimated $8.9 billion reaching 10% of this population, $4.5 billion reaching 5%, and 0.9 billion reaching 1%. If assuming $583.33 per participant cost, national healthcare savings were estimated at $2.4 billion if the program reached 10% of this population, $1.2 billion reaching 5%, and $0.2 billion reaching 1%.

## Discussion

This study reaffirms the importance and potential of community-based self-management interventions rooted in public health to control healthcare costs among adults with chronic conditions. Extrapolating the estimated $364 cost saving per CDSMP participant results in meaningful national savings (i.e., ~$3.3 billion), if program penetration reaches only 5% of all individuals with one or more chronic condition. Assuming a $350 average CDSMP cost per participant, we could achieve potential national healthcare savings from $0.7 billion to $65.7 billion by averting from ER visits and hospitalizations if CDSMP reaches a minimum of 1% of adults having at least 1 chronic condition (i.e., the range of national savings depends on the level of program penetration). The cost savings achieved herein among heterogeneous populations served by diverse organizations were substantial; however, they were slightly lower than those estimated in the original, more tightly controlled, randomized trial which included controls whose hospital-related healthcare costs increased [4].

Previous studies have documented the value of CDSMP in improving participants’ health behaviors, disease-related symptoms, communications with providers, and overall health status [4,5]. With the addition of findings from the current study, it is clear that this intervention can influence all aspects of the Triple Aim (i.e., enhanced care, improved health, and better value) [6,13]. Modest past investments by the U.S. Administration on Aging, the Centers for Disease Control and Prevention, and other agencies have established a viable foundation for scaling up this intervention. Within the past 5 years, over 150,000 people have participated in CDSMP workshops through the Communities Putting Prevention to Work Initiative and other public-private collaborations [12]. This highlights the probability of high-level CDSMP penetration to reach populations with chronic conditions as long as strong support and funding sources are available for this initiative. As such, additional public sector resources are needed to continue the momentum and leverage the existing infrastructure. At the same time, private insurers are highly encouraged to provide benefits to their patients with chronic conditions by discounting their premiums as well as CDSMP workshop delivery agencies by providing generous reimbursements.

A policy issue of interest surrounds how efficiencies in CDSMP workshop delivery can increase overall savings. In the current study, we have used an estimated $350 per participant cost in our calculations assuming 10 participants in a workshop and $3,500 workshop costs. To estimate this average cost, we relied on experts’ opinions including the program developers, field reports (ranging from $204 to $375) [14,15], and an unpublished survey among state CDSMP implementers conducted by the National Council on Aging (ranging from $150 to $750) [16]. However, it is worth noting that the cost estimation should vary by the number of CDSMP participants in a workshop and the administrative capacity of the delivering agencies. Nevertheless, we would expect costs to decrease with the efficiencies gained through capacity building accompanying widespread dissemination. A prior study projected lower average CDSMP costs for agencies with higher numbers of participants over time compared to the other agencies with lower numbers of participants, which emphasizes the importance of strong recruitment efforts and collaborating with community partners [15]. Further studies are warranted to identify how costs associated with marketing and administration would be affected when scaling up for widespread program delivery.

It is important to ensure that the cost-saving benefits of CDSMP equitably reach various populations despite geographic location and demographic factors. The small enrollment in some workshops highlights potential difficulties of scaling up in rural areas as these areas typically have smaller class sizes due to population dispersion and lack of infrastructure supports. However, on the positive side, CDSMP is being widely disseminated throughout the United States (e.g., between 2010 and 2012 more than 100,000 participants enrolled in CDSMP programs sponsored by the Administration on Aging) [12]. A prior study analyzing demographic factors and disease profiles among more than 100,000 CDSMP participants (between 2010 and 2012) documented how representative CDSMP participants are of the adult population [12]. When comparing to 2010 U.S. Census, CDSMP participants tended to be more female (77.7%) and older (mean age = 67 years) compared to the Census (51%, 37 years) [11]. However, there were similarities in terms of rural residence and race/ethnicity. Approximately 25% of CDSMP participants resided in rural areas (compared to 19.3% of Census) and had a similar racial/ethnic composition (white of CDSMP: 66.4% vs. 63.7% of Census; African American: 21.5% vs. 12.2%; Hispanic: 17.0% vs. 16.3%; Asian/Pacific Islanders: 4.5% vs. 4.9%; American Indians: 1.6% vs. 0.7%) [17]. These statistics are encouraging, especially when considering the capacity of evidence-based interventions to reach various populations at risk of chronic conditions.

Additionally, the potential of CDSMP to contribute to cost savings while improving health status provides a strong incentive for alignment with Accountable Care Organizations, models of enhanced primary care, initiatives for dually eligible beneficiaries, and State Innovation Models. To better integrate and leverage CDSMP to improve healthcare organization and financing, new initiatives are needed to design and test ways to: 1) strengthen collaboration among healthcare organizations, community partners, and public health agencies; 2) establish useful quality measures related to self-management; and 3) incentivize providers to further support evidence-based approaches to self-management.

### Study limitations

First, data were drawn from a national study with a pre-post design appropriate for addressing translational research questions. While the current study lacks a comparison group, improvements were generally similar as those reported in the original randomized trial with some attenuated cases [4]. Our current study design does not permit the elimination of potentially confounding factors influencing study outcomes. Second, healthcare utilization was self-reported resulting in the possibility of recall bias. Nevertheless, a prior study found high concordance between self-reported and objectively measured ER visits and inpatient use [18]. Third, the current 12-month study may require a longer study duration to conclude definitively the healthcare cost-saving effects of CDSMP; however, we expect to see sustainable effects of reducing ER visits based on prior 2-year study [4]. Last, the current study is based on a critical assumption that we can extrapolate the healthcare cost savings of the National CDSMP to the national level using census data. Therefore, the cost-saving effects of CDSMP should be further studied to account for demographic changes in the Americans population over time and variations by population’s disease profiles. Nevertheless, we caution that it will be difficult to accurately estimate cost-saving effects by specific chronic condition types given the presence of multiple chronic conditions and the multitude of different disease clusters. To provide context, a previous study reported that CDSMP participants have on average 2.2 chronic conditions (e.g., hypertension = 43.0%; arthritis = 40.8%; diabetes = 30.3%; depression = 19.5%) [12]. Future study is needed to examine the average per participant cost of CDSMP based on geographic locations and capacities of agencies to deliver CDSMP.

## Conclusion

Future efforts should explore these issues in light of the complexities of estimating costs and cost savings from existing field studies. However, the fundamental findings of the current study (i.e., the potential of CDSMP type programs to accrue cost savings through decreased ER visits and hospitalizations) suggest that evidence-based self-management programs are cost-saving and health-enhancing strategies for dealing with the epidemic of chronic conditions, especially with workshops with 10 or more participants. We recommend immediate attention be given to initiate system changes and policies that increase the awareness of self-management programs among patients as well as physicians, support the development of a delivery infrastructure, and help defray the costs of widespread dissemination of such programs. CDSMP delivers wide range of important outcomes, with a return on investment of 1:1 – which means this tertiary prevention intervention provides substantial value, more than paying for itself.

## Abbreviations

CDSMP: Chronic disease self-management program; ER: Emergency room; MEPS: Medical expenditure panel survey.

## Competing interests

The authors declare that they have no competing interests, with one exception that we are disclosing. Kate Lorig, the program developer, receives royalties from the book used by participants in the CDSMP program. However, independent analyses were conducted on all study outcomes by Texas A&M Health Science Center.

## Authors’ contributions

SA planned the study, analyzed the data, and wrote the article. RB analyzed the data and wrote the article. MLS assisted in data interpretation and in critical revision. LJ analyzed the data. KL assisted in data interpretation and provided critical revisions. NW provided critical revisions. MGO conceived and supervised the study, and provided critical content and revisions. All authors read and approved the final manuscript.

## Pre-publication history

The pre-publication history for this paper can be accessed here:

http://www.biomedcentral.com/1471-2458/13/1141/prepub

## References

[B1] Harris JR Wallace RB The institute of Medicine’s New report on living well with chronic illness Prev Chronic Dis 2012 13 E148 DOI: 10.5888/pcd9.120126 22995102 10.5888/pcd9.120126 PMC3475533

[B2] Anderson GF Chronic care: making the case for ongoing care 2010 Princeton, NJ: Robert Wood Johnson Foundation

[B3] National Prevention Council National prevention strategy 2011 Washington, DC: US Department of Health and Human Services, Office of the Surgeon General

[B4] Lorig KR Ritter P Stewart AL Sobel DS Brown BW Jr Bandura A Gonzalez VM Laurent DD Holman HR Chronic disease self-management program: 2-year health status and health care utilization outcomes Med Care 2001 13 11 1217 1223 10.1097/00005650-200111000-00008 11606875

[B5] Brady TJ Murphy L Beauchesne D Bhalakia A Chervin D Daniels B Greenberg M House M O’Colmain B Sorting through the evidence for the Arthritis Self-Management Program and the Chronic Disease Self-Management Program: Executive Summary of ASMP/CDSMP meta-analysis 2011 Atlanta: Centers for Disease Control and Prevention http://www.cdc.gov/arthritis/docs/asmp-executive-summary.pdf

[B6] Ory MG Ahn S Jiang L Smith ML Ritter P Whitelaw N Lorig K Successes of a national study of the chronic disease self-management program: meeting the triple Aim of health care reform Med Care 2013 13 11 992 998 10.1097/MLR.0b013e3182a95dd1 24113813

[B7] Ory MG Ahn S Jiang L Lorig K Ritter P Laurent D Whitelaw N Smith ML National study of chronic disease self-management: six month outcome findings J Aging Health 2013 13 7 1258 1274 10.1177/0898264313502531 24029414

[B8] Whitelaw N Lorig K Smith ML Ory MG National Study of Chronic Disease Self-Management Programs (CDSMP) 2013 from http://www.ncoa.org/cha

[B9] Lee AH Zhao Y Yau KKW Xiang L How to analyze longitudinal multilevel physical activity data with many zeros? Prev Med 2010 13 6 476 481 10.1016/j.ypmed.2010.09.012 20920520

[B10] Agency for Health Research and Quality (AHRQ) Medical Expenditure Panel Survey Household Component: 2010 Full Year Consolidated Data File 2010 Rockville, MD http://meps.ahrq.gov/mepsweb/data_stats/download_data_files_detail.jsp?cboPufNumber=HC-138

[B11] Howden LM Meyer JA Age and sex composition: 2010 2010 Census Briefs, US Department of Commerce, Economics and Statistics Administration US Census Bureau 2010 Washington, DC Retrieved May 10, 2013, from http://www.census.gov/prod/cen2010/briefs/c2010br-03.pdf

[B12] Ory MG Smith ML Patton K Lorig K Zenker W Whitelaw N Self-management at the tipping point: reaching 100,000 Americans with evidence-based programs J Am Geriatr Soc 2013 13 5 821 823 10.1111/jgs.12239 23672545

[B13] Berwick DM Nolan TW Whittington J The triple aim: care, health, and cost Health Aff 2008 13 3 759 769 10.1377/hlthaff.27.3.759 18474969

[B14] Bovbjerg VE Kingston MSJ Program Impact Report: Oregon’s living Well with Chronic Conditions 2010 Oregon State University College of Health and Human Services Retrieved May 11, 2013, from [http://public.health.oregon.gov/DiseasesConditions/ChronicDisease/LivingWell/Documents/Living%20Well%20Program%20Impact%20Report%20Final.pdf]

[B15] Page TF Palmer RC Cost analysis of chronic disease self-management programmes being delivered in South Florida Health Educ J 2013 10.1177/0017896912471047

[B16] National Council on Aging Chronic Disease Self-Management Education Integrated Services Delivery System Assessment Tool 2013 Washington, DC http://www.ncoa.org/improve-health/center-for-healthy-aging/content-library/CDSME-Sustainability-Tool.pdf

[B17] US Census Bureau United States Census 2010 2010 Washington, DC: US Department of Commerce Retrieved May 15, 2013, from [http://www.census.gov/2010census/]

[B18] Ritter PL Stewart AL Kaymaz H Sobel DS Block DA Lorig KR Self-reports of health care utilization compared to provider records J Clin Epidemiol 2001 13 2 136 141 10.1016/S0895-4356(00)00261-4 11166528

